# The YAP/TAZ Signaling Pathway in the Tumor Microenvironment and Carcinogenesis: Current Knowledge and Therapeutic Promises

**DOI:** 10.3390/ijms23010430

**Published:** 2021-12-31

**Authors:** Ángel Ortega, Ivana Vera, Maria P. Diaz, Carla Navarro, Milagros Rojas, Wheeler Torres, Heliana Parra, Juan Salazar, Juan B. De Sanctis, Valmore Bermúdez

**Affiliations:** 1 Endocrine and Metabolic Diseases Research Center, School of Medicine, University of Zulia, Maracaibo 4004, Venezuela; angelort94@gmail.com (Á.O.); ivanaa19.iv@gmail.com (I.V.); mariadiazalbornoz@hotmail.com (M.P.D.); cpnm24@gmail.com (C.N.); migarocafi@gmail.com (M.R.); wheelertorres@gmail.com (W.T.); helianapp20@hotmail.com (H.P.); juanjsv18@hotmail.com (J.S.); 2 Institute of Molecular and Translational Medicine, Faculty of Medicine and Dentistry, Palacký University Olomouc, 77900 Olomouc, Czech Republic; sanctisj@gmail.com; 3 Universidad Simón Bolívar, Facultad de Ciencias de la Salud, Barranquilla 080002, Colombia

**Keywords:** YAP/TAZ, TEAD, Hippo signaling pathway, carcinogenesis, tumor microenvironment, neoplastic stem cells, cell proliferation, drug resistance, chemoresistance, immunotherapy

## Abstract

The yes-associated protein (YAP) and the transcriptional coactivator with PDZ-binding motif (TAZ) are transcriptional coactivators, members of the Hippo signaling pathway, which play a critical role in cell growth regulation, embryonic development, regeneration, proliferation, and cancer origin and progression. The mechanism involves the nuclear binding of the un-phosphorylated YAP/TAZ complex to release the transcriptional enhanced associate domain (TEAD) from its repressors. The active ternary complex is responsible for the aforementioned biological effects. Overexpression of YAP/TAZ has been reported in cancer stem cells and tumor resistance. The resistance involves chemotherapy, targeted therapy, and immunotherapy. This review provides an overview of YAP/TAZ pathways’ role in carcinogenesis and tumor microenvironment. Potential therapeutic alternatives are also discussed.

## 1. Introduction

Cancer continues to be the second most frequent cause of death worldwide, with an estimated 10 million deaths in 2020 [1]. Carcinogenesis is a process in which multiple stages exist in synergy and are intimately related. This process leads to the formation of malignant cells capable of excessive proliferation and dissemination [2]. It was assumed that these unique cellular characteristics are acquired due to the successive accumulation of changes in the genetic material. However, it was later discovered that aside from chemical or genetic alterations, the surrounding environment is essential to trigger the evolution of malignant neoplasia at the tissue level [3]. Cellular intercommunication plays a critical role in cancer origin, progression, recurrence, and relapse [4].

Tumor progression usually involves the overactivation of signaling pathways [5] related to cell growth and behaviour involved in cancer development [6]. One of the most important signaling pathways is the Hippo pathway, a tumor suppressant pathway that inhibits excess proliferation and controls organ size and apoptosis evasion [7]. This pathway is inhibited in certain types of cancer [8].

The critical steps of the Hippo pathway are represented by the yes-associated protein (YAP) and the transcriptional coactivator with PDZ-binding motif (TAZ) [9]. This complex is phosphorylated in the cytoplasm and unphosphorylated in the nucleus. Upon dephosphorylation, the YAP/TAZ complex translocates to the nucleus. Once in the organelle, the complex binds preferentially to the transcriptional enhanced associate domain (TEAD) to release them for its repressors [10]. The complex is then responsible for cell proliferation and transformation. Abnormal nuclear expression of YAP/TAZ proteins is associated with poor prognosis of different types of cancer [11,12,13,14,15]. In addition, YAP/TAZ can associate with other transcription factors leading to cell remodelling, proliferation, and invasion [16]. Therefore, this review aims to summarize the role of YAP/TAZ pathways in carcinogenesis and the tumor microenvironment and highlight the importance of potential as therapeutic alternatives involving this signaling pathway.

## 2. YAP/TAZ Structure and Function

YAP and TAZ are cotranscriptional coactivators with alternating locations, the cytoplasm and the nucleus [17]. Both have a similar structural composition, sharing over half of their amino acid sequences and characteristics. YAP and TAZ are multidomain proteins that interact with several other proteins, and their WW domains allow them to bind, for example, to proteins containing a PPxY motif. They also interact with TEAD through regions that have been mapped within their N terminal, and they promote oncogenic transformation in some cancers [18].

The *YAP* gene is located in the 11q22 chromosome in humans. The central region of the protein comprises: (1) one or more WW domains, (2) a transactivation domain on its C-terminal region, (3) a double spiral domain, and (4) a PDZ-binding motif. This gene has eight isoforms classified into two large groups: YAP1 and YAP2. The product differs in the number of WW domains.

The *TAZ* gene is located in the 3q23-q24 chromosome, and it is abundantly expressed in different tissues. There are three variants in humans formed by one WW domain. Unlike YAP, TAZ does not have an Src homology 3 (SH3) binding motif nor a double spiral domain [19].

**Figure 1:**
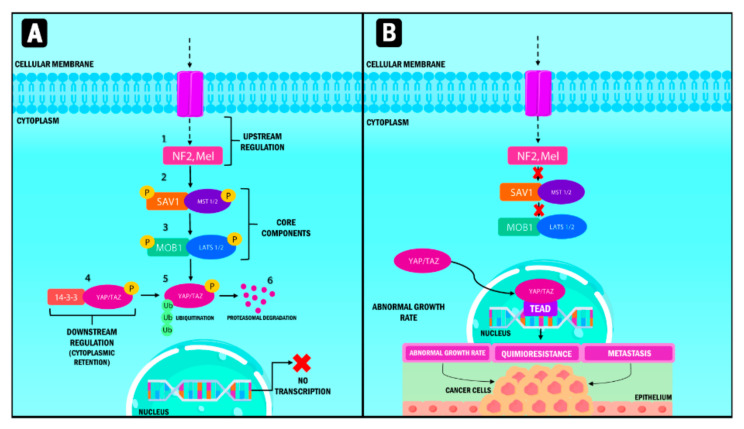
Representation of the Hippo signaling pathway in humans. When the Hippo pathway is active (**A**), multiple signals stimulate the upstream components, which are NF2 and Mel. Subsequently, MST 1/2 protein and its substrate, LATS 1/2, are activated through phosphorylation processes and phosphorylate YAP/TAZ, which interacts with the 14-3-3 protein. Phosphorylation causes their cytoplasmic retention and generates ubiquitination processes mediated by proteasome degradation. When the Hippo pathway (**B**) is inactive, YAP/TAZ that are not phosphorylated translocate into the nucleus and form a transcription factor complex by binding to TEAD. This pathway regulates the transcription of genes involved in abnormal multiplication, chemoresistance and metastasis. YAP: Yes-associated protein; TAZ: Transcriptional coactivator with PDZ-binding motif; NF2: Neurofibrominin 2; MST: Mammalian sterile 20-like kinase; LATS: large tumor suppressor kinase; SAV1: Protein salvador homolog 1; MOB1: Mps1-binder-related; TEAD: Transcriptional enhanced associate domain.

YAP/TAZ and the Hippo pathway play an essential role in cell and tissue physiology in normal tissue. However, they are also crucial in cancer origin and progression (Figure 1) [20]. Three fundamental components are involved in their up-regulation: (1) neurofibromin 2 (NF2) and Mel, the nucleus components; (2) kinase 1/2 proteins, including the sterile 20-like kinase 1 and 2 (Mst1, Mst2), and (3) the large tumor suppressor kinase (Lats 1/2).

Finally, the down-regulated pathways [21] are divided into a serine-threonine kinase regulator module (formed by the nucleus components); and a transcriptional module in which both YAP and TAZ are present. Both modules are closely related, interacting to perform inverse functions since the kinase module is a tumor suppression factor, while the transcriptional module corresponds to the oncogenes [22].

In physiological conditions, the functions of the tumor suppressant module are the ones that prevail. The pathway starts with the stimulation of Mst 1/2 proteins through different signals that activate its Lats 1/2 substrates through a phosphorylation process. Lats 1/2 phosphorylate YAP/TAZ molecules that interact with the 14-3-3 protein once activated, resulting in their cytoplasmic retention [19,23]. Consequently, phosphorylated YAP/TAZ is ubiquitinated and degraded by the proteasome [24].

## 3. Role of YAP/TAZ in Carcinogenesis

When the functions of their transcriptional module are manifested, YAP/TAZ induces the expression of genes that favor transcription and anti-apoptotic genes through their interaction with transcriptional factors [25]. This event results in the appearance of cancer hallmarks such as the induction of proliferative signaling, activation of therapeutic resistance and cell death evasion and metastasis [26,27]. Since YAP/TAZ does not have binding domains to the DNA molecule, it must form complexes with DNA-binding proteins to regulate gene expression. Various transcriptional factors have been identified to interact with YAP/TAZ, such as Activator protein-1 (AP-1), E2F, MYC, myocardin-related transcription factor/serum response factor (MRTF/SRF), RUNX, Transcriptional Repressor GATA Binding 1 (TRPS1), Zinc Finger E-Box Binding Homeobox 1 (ZEB1), SNAIL, SLUG, Notch Intracellular Domain/Recombination Signal-Binding Protein for Ig Kappa J region (NICD/RBPJ), among several others [16]. However, the TEAD appears to be the most critical partner for transactivation and growth promotion [28].

It has been determined that YAP/TAZ interacts with the chromatin-remodeling SWI/SNF complex components through their WW domain. YAP/TAZ mediates the union or closeness of the methyltransferase complex and the transcription factor. This event causes the acceleration of histone methylation and the transcription of the target genes [29]. The cell then acquires characteristic cancer stem cells (CSC) properties, such as abnormal multiplication, chemoresistance, and metastasis [30]. An immunohistochemical study performed by Debaugnies et al. revealed that the expression of YAP/TAZ is a critical determinant in the early stages of base cell carcinoma (BCC) and squamous cell carcinoma (SCC). The study used mice models and human tissue for each type of cancer, and the results established that YAP/TAZ expression was consistently active in both study models.

Furthermore, it was found that YAP/TAZ was required for the immediate survival of the cells expressing oncogenes [31]. Likewise, in a study performed by Hagenbeek et al., the role of YAP/TAZ in liver cancer and the distinctive role of each molecule was established. It was observed that YAP expression is amplified in liver cancer, promoting carcinogenesis by inhibiting tumor suppressant kinases in an inflammatory and damaged tissue milieu. TAZ also stimulates the production of inflammatory cytokines and the infiltration of macrophages into the tissue. The intimate relationship between cancer and inflammation led to the theory that TAZ could induce inflammation and promote the oncogenic activity of YAP [32]. Thus, aberrant expression of these transcription cofactors is related to the genesis and growth of cancer cells [33].

The effects of the YAP/TAZ molecules are not limited to the Hippo pathway. However, they are considered an integrator nexus in multiple signaling pathways that control the formation and progression of cancer [9]. One of these is the Wnt pathway, which is integrated with the Hippo pathway. In a study performed by Park et al., mice were used to analyze the role of YAP/TAZ in the non-canon or alternative Wnt pathway. It was reported that YAP/TAZ molecules are critical mediators in the Wnt pathway through their activation performed by Wnt5a/b and Wnt3a. The authors defined this as the “alternative Wnt-YAP/TAZ signaling axis” resulting in TEAD-mediated transcription [34].

In addition, Lim et al., demonstrated that Wnt signaling promotes breast cancer by blocking YAP/TAZ ITCH-mediated degradation in the breast tissue [35]. Bisso et al., also performed a study in which the *Myc* oncogene and the Wnt/catenin signaling pathway were conditionally activated in liver cancer. The authors concluded that YAP/TAZ accumulated through the activation of the previously mentioned elements and are responsible for the proliferative cell response and cell growth and survival [36], cell proliferation plasticity, and competition [37].

As key effectors on the Hippo pathway, YAP/TAZ molecules control cell growth and the size of the organs. Therefore, their deregulated expression leads to carcinogenesis as they control the expression of different cell cycle regulators and factors involved in DNA repair and mitosis [38]. AP-1, involved in mitosis, and the YAP/TAZ-TEAD complex cooperate and participate in the growth and proliferation of tumor cells in breast cancer, liver cancer and uveal melanoma [39].

YAP/TAZ can also potentiate proliferation in the carcinogenesis process by using transcriptional factors that amplify their functions through a feedforward activation loop [40]. Such is the case of the *NUAK2* gene, identified as a direct inhibitor of the kinase module that belongs to the Hippo pathway. This inhibition of the pathway ensures the activation of YAP/TAZ leading to tumor growth [41]. In addition, YAP/TAZ modulates cell metabolism, glycolysis, glutamine metabolism, lipogenesis and cholesterol synthesis. This way, the nutrients needed to maintain proliferation during carcinogenesis are ensured [42].

Cancer cells have the potential of modifying their fates depending on their needs through plasticity [43]. Plasticity includes processes such as dedifferentiation (reversion of specialized structures) and transdifferentiation (reprogramming) [44,45]. YAP/TAZ favors cell plasticity for their proliferation, progression, and dissemination [46]. A study performed by Panicerra et al. showed that YAP/TAZ could reprogram different cell lineages in their respective tissue-specific stem cells. This effect indicates that YAP/TAZ induces the creation of new CSC from normal cells. However, the mechanisms through which they achieve this transition continues to be an enigma [47].

The importance of TAZ in cell plasticity is well recognized since it is required for the autorenewal of cells and the start of tumor growth in breast cancer. TAZ provides CSC properties to normal cells, making them capable of originating chemo-resistant tumors [48]. Cancer cells with different characteristics are compared with those surrounding them during cell competition to determine their survival and expansion [49]. In this interaction, one cell population survives while the other is extinguished. It is essential to highlight that the result of this competition is not an autonomous process of the cell but results from the dynamic interaction of the cells involved in which the ones who emerge victorious from this competition completely overtake the tissue territory. At the same time, those who lose are excluded from the apical region to face senescence or apoptosis processes [50].

Recent studies have demonstrated that YAP/TAZ are protagonists in this process, especially the YAP molecule [49,51]. Liu et al. [52] found that YAP1 expression at different cell lines from glioblastoma tumors leads to cells expressing higher levels of this factor and growing faster than the rest. This way, carcinogenesis and clonal dominance are promoted. Consequently, oncogenic lesions are used by tumors to their benefit, involving the cancer cells surrounding them in competitive processes to ensure their survival and progress [52].

## 4. YAP/TAZ and the Tumor Microenvironment

Solid tumors are surrounded by a complex and heterogeneous microenvironment formed by numerous components that directly or indirectly contribute to maintaining their survival [53]. The pro-tumor microenvironment involves (1) acellular components, the extracellular matrix (ECM), exosomes, and cytokines, and (2) cellular components, fibroblasts, endothelial cells, adipocytes, and immune cells [54]. In addition, it is characterized by acid pH, hypoxia, increased interstitial pressure, and fibrosis [55]. These elements create complex interactions between cancer cells and the stromal components of the microenvironment to create a positive feedback loop that promotes cancer development [56].

**Figure 2:**
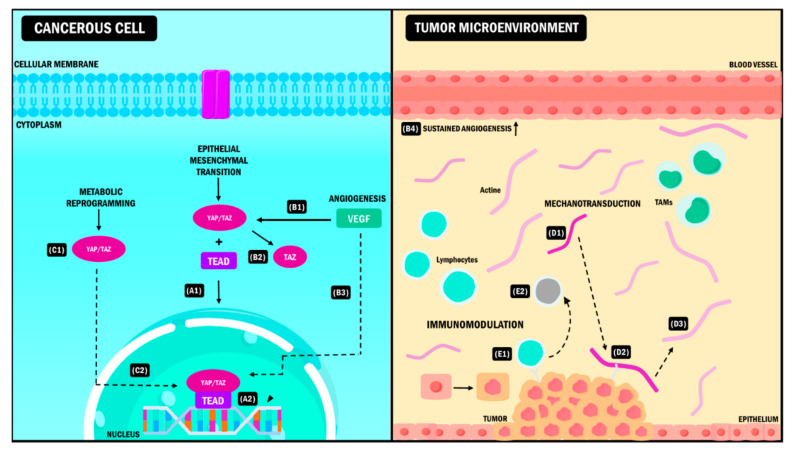
YAP/TAZ signaling pathway in the tumor microenvironment. YAP/TAZ coactivators interact simultaneously with different elements of the TME causing (**A**) EMT during neoplastic progression. After the coupling of YAP/TAZ with the TEAD (**A1**), the complex is activated, and it induces the expression of target genes (**A2**) that promote the transition from the epithelial phenotype to the mesenchymal phenotype. (**B**) Angiogenesis. The presence of VEGF (**B1**) stimulates the activation of TAZ (**B2**) and the subsequent expression of genes (**B3**) that promote sustained angiogenesis of the blood vessels in the TME (**B4**). (**C**) Metabolic reprogramming. YAP/TAZ activation and the formation of the YAP/TAZ/TEAD complex (**C1**) promotes the expression of target genes that allow the cancer cell to acquire new metabolic pathways needed for its adaptation to the TME (**C2**). (**D**) Mechanotransduction. During tumor progression, the EMC adopts a rigid conformation (**D1**). YAP/TAZ act as sensors that detect EMC changes due to adhesion molecules (**D2**), promoting modifications in TME properties and intrinsic changes in tumor cells that allow them to survive in these conditions (**D3**). (**E**) Immunomodulation. When YAP/TAZ interact with immune cells (**E1**), they induce changes in the antitumoral phenotype of these cells promoting immune evasion, which leads to neoplastic progress (**E2**). YAP: Yes-associated protein; TAZ: Transcriptional coactivator with PDZ-binding motif; TME: Tumor Microenvironment, VEGF: Vascular Endothelial Growth Factor, EMT: Epithelial-Mesenchymal Transition, EMC: Extracellular Matrix, TAMs: Tumor-Associated Macrophages, TEAD: Transcriptional enhanced associate domain.

In this order of ideas, YAP/TAZ molecules are actively expressed in CSC. They represent critical constituents for their expansion from the differentiation of normal cells to cells with malignant characteristics [57]. Therefore, YAP/TAZ molecularly disrupt the cell populations to begin the formation of tumors. Other additional functions include favoring the secretion of growth factors that induce angiogenesis in epithelial cells [58] and increasing the expression of molecules that chemo-attract myeloid-derived suppressor cells (MDSCs) [59]. Finally, they also promote proinflammatory cytokine production in the fibroblasts associated with the tumor, favoring a rigid ECM [30,60]. All these functions are vital in forming the tumor microenvironment (Figure 2) [61].

## 5. Remodelling of the Tumor Microenvironment

### 5.1. Epithelial-Mesenchymal Transition

Epithelial to mesenchymal transition (EMT) involves a cell program with the main function of regulating the different stages of embryo morphogenesis [62]. It confers cells the ability to lose their cell adhesions, apical and basal polarity, and favors transdifferentiation to a mesenchymal phenotype [63]. This transition allows tumor cells to complete many of the invasion and metastasis cascade stages during tumor progression. It also influences the acquisition of resistance to apoptosis, blocks cell senescence, increases survival, facilitates genomic instability, alters metabolism and induces suppression of the immune system [64].

In cancer, this process is transient and may be reversible [65]. After the invasion and completion of the metastatic cascade, tumor cells undergo reciprocal changes that revert the mesenchymal phenotype to an epithelial phenotype where tumor cells obtain apical and basal polarity, reorganize their cytoskeleton and recover their cellular adhesions. This process is known as mesenchymal to epithelial transition (MET) [66,67].

However, both transitions are not the result of simple changes between two rigid phenotypes; therefore, when observing the behavior of tumor cells it is evident that they acquire phenotypes and mesenchymal characteristics without altogether abandoning their epithelial features [68]. This is known as “epithelial-mesenchymal plasticity” (EMP) and it grants cancer cells the ability to navigate between three distinctive phenotypes: mesenchymal, epithelial and hybrid, depending on the type of tissue and their needs [67].

Among all the pathways and genes involved in the EMT, YAP/TAZ are the master coordinators of this process [69,70]. The exact mechanism through which they perform this role remains uncertain. It has been described that during cancer, EMT mediated by these coactivators starts with the coupling of the YAP/TAZ/TEAD complex at the cell nucleus. This event leads to the transcription of EMT mediators and factors that allow tumor cells to convert to a stem cell with pluripotential phenotype. Notably, the amplification of their effects gives YAP/TAZ the ability to be potent inductors of this biological program. In a study performed by Diepenbruck et al., an increase in the expression of TEAD2 in EMT was observed. It is suggested that this could lead to the formation of YAP/TAZ and the translocation of these cofactors, and their increase would be enough to induce EMT and metastasis in lung cancer [71].

During EMT, the simultaneous activation of both cofactors is not instrumental, which was shown in the study performed by Zhou et al. It was reported that only YAP1 is responsible for the transition in gastric carcinoma. This effect was observed by analyzing different cell populations from gastric carcinoma tissues. EMT induction occurs through the *SOX9* gene, responsible for activating the YAP1 signaling pathway [72]. Conversely, research performed by Chen et al. showed evidence that TAZ overexpression promotes EMT in ovarian cancer. This overexpression was observed by analyzing mRNA expression in seven ovarian cancer tissue samples. The results showed that TAZ was expressed in five of the seven tumor samples. Thus, the molecule is required for these types of cancer [73].

In this regard, YAP/TAZ act as a regulatory center for signals that integrate various biophysical and biochemical stimuli responsible for their phosphorylation and effectiveness in hybrid EMT/MET [74,75]. A representation of this is the expression of YAP/TAZ in Ewing’s Sarcoma, which has a homogeneous origin. However, it derives from cells with heterogeneous features where the EMT/MET process is reversible [76]. During the invasion, YAP/TAZ regulate the expression of proteins secreted by the ECM, proteoglycan 4 (PRG4) and tenascin C (TNC). Both molecules produce their nuclear translocation and the activation of target genes, thus promoting EMT; however, at the end of the process the transcriptional factor EWS-FLI1 occupies and blocks the YAP/TAZ/TEAD binding sites, thus interfering with the genetic programming of EMT [77].

This suggests that although YAP/TAZ are avid coordinators of EMT, they are not necessarily responsible for the reversion of tumor cells to an epithelial phenotype. However, in turn both are indispensable for EMT and required for MET [78,79]. In this sense, the evidence presented indicates that YAP/TAZ can induce changes in the phenotype of tumor cells and produce EMT depending on their gene expression levels and cellular context, creating a bidirectional link between the activation of these transcription cofactors and the maintenance of the EMT genetic program.

### 5.2. Angiogenesis

Angiogenesis corresponds to the creation of new blood vessels from the existing vasculature [80]. It is a mechanism used by cancer cells to access the nutrients they need, ensure their survival and eliminate waste products [81]. It has been established that genes such as *YAP/TAZ* can regulate the angiogenesis process during tumor progression [82]. YAP/TAZ participation in the angiogenesis process is performed through different mechanisms, of which the role of the vascular endothelial growth factor (VEGF) is the most significant [83]. VEGF can stimulate and promote angiogenesis in tumor cells and directly affect the maintenance of CSC [84]. According to recent data from breast cancer studies, VEGF can specifically activate the TAZ molecule, producing CSC renewal through this interaction and provoking sustained angiogenesis [85,86].

Xu et al., performed a study in which they found evidence that VEGF activates TAZ in astrocytomas. Moreover, the non-regulated expression of both components could be responsible for the aberrant proliferation of this type of cancer. In vitro studies observing the role of YAP in angiogenesis revealed that the molecule increases this process in kidney cell carcinoma, promoting the expression and secretion of VEGF through Gli2 [87].

Different types of ligands and receptors belonging to multiple signaling pathways can form functional networks. These networks play a critical role in promoting and maintaining angiogenesis [88]. Notch ligands Delta-like 4 (DLL4)/Notch, angiopoietin/TIE2, Wnt/frizzled, platelet-derived growth factor/platelet-derived growth factor receptor (PDGF/PDGFR), and the integrin/cell-matrix belong to these signaling pathways that are intimately related to YAP/TAZ. Therefore, both can be considered vital cell molecules activated by multiple signals to modulate angiogenesis [89,90,91].

### 5.3. Mechanotransduction

The mechanotransduction process is the biochemical signal generated by cells upon a mechanical stimulus from the microenvironment. The consequence of mechanotransduction is tumor growth, differentiation and progression [92]. This abnormal cell behavior is mainly related to the increased rigidity of the ECM in tumors, which causes the loss of cell polarity and, consequently, the migration and metastasis of tumor cells [93].

YAP/TAZ have been identified as sensors and mediators of mechanical signals from the microenvironment. They are responsible for the mechanotransducer and mechanosensory characteristics in the tumour tissue [46]. ECM rigidity controls the location of YAP/TAZ in the cell and when a soft ECM surrounds the cells, the molecules are located in the cytoplasm and remain inactive. However, when there is a rigid ECM, the molecules are located in the nucleus, promoting gene transcription [94].

It has been proposed that the increased contractibility of the tumor cell is due to the binding of cell actomyosin to ECM and the rigid complex induces the transcription of YAP/TAZ [95]. This mechanism begins with the adhesion of cells to the ECM through integrin receptors and cell junctions that mediate the transmission of signals between the ECM and the F-actin cytoskeleton. The increased interactions between ECM and focal adhesion promotes YAP/TAZ activity. Studies have demonstrated that the “knockdown” of the F-actin-capping protein subunit beta (Capzb) results in the increase of the amounts of nuclear YAP/TAZ and their transcriptional activation [96,97]. F-actin affects YAP/TAZ activity on the Hippo pathway through the dependent or independent phosphorylation of LATS1/2 [98]. Simultaneously, they cause stress from the mechanical activation response mediated by the cytoskeleton [99].

The mechanotransduction mediated by YAP/TAZ causes ECM rigidity in tumors, mainly found in breast cancer. Its activation results in the production of laminin 511 and autocrine activation. Integrins are associated with the maintenance of tumor oncogenic activity [100]. Likewise, it is essential to highlight the relevance of YAP activation in the fibroblasts in breast cancer tissue, as this can promote rigidity in the ECM, the invasion of tumor cells and angiogenesis.

Calvo et al. showed that YAP activation is different in fibroblasts. YAP induces remodeling of the ECM by activating the actomyosin cytoskeleton and expressing other factors such as Anillin Actin Binding Protein (ANLN) and Diaphanous Related Formin 3 (DIAPH3). These establish a feedforward self-reinforcing loop that maintains the phenotype of the fibroblasts in the microenvironment as well as greater matrix rigidity, resulting in a non-favorable prognosis for the patients [101].

### 5.4. Stromal Immunomodulatory Responses

One of the immunomodulatory effects of YAP/TAZ is the direct transcriptional expression of programmed death-ligand 1 (PD-L1) in tumor cells [102]. In malignant neoplasia, tumor cells use PD-L1 expression to inhibit cell responses [103]. Recent evidence reported by Rensburg et al., as well as Lee et al., demonstrated that YAP/TAZ expression regulates, intensifies, and at the same time increases PD-L1 levels in breast and lung cancer. In the first study, ligand expression in tumor cells was determined by the union of the TAZ molecule to the PD-L1 promoter through the TEA domain. This binding results in the disruption of T cells. In the second study, genes that significantly increase in cell populations expressing PDL-1 are conserved through the overexpression of TAZ [104,105]. Results similar to those previously described were found in studies focusing on other types of cancer, such as malignant pleural mesothelioma and non-small cell lung cancer (NSCLC) [106].

The YAP/TAZ complex is associated with the recruitment and the activity of different immune cells in the tumor microenvironment, such as tumor-associated macrophages (TAMs) and MDSCs. It has been reported that YAP expression is directly linked to the polarization of TAMs to the M2 phenotype [107]. This polarization to M2 reduces the capacity of antigenic presentation. Moreover, M2 macrophages secrete immunosuppressive factors, IL-10, transforming growth factor β (TGF-β), and C-C motif chemokine 22 (CCL22). It also inhibits the cytotoxicity of T-cells through the PD-L1/PD-1 signaling axis [108]. These effects suggest that the immunoregulator effects of YAP/TAZ regarding the expression of this ligand transcend their direct relationship with tumor cells. Thus, YAP/TAZ are versatile, avid, and multifunctional regulators as well as transcriptional coactivators.

Zao et al., reported that the Nogo B modulator promotes the polarization of the TAMS to the M2 phenotype in hepatic cell carcinoma, inducing the YAP/TAZ signaling pathway and promoting tumor angiogenesis, invasion, and metastasis [109]. Similarly, Huang et al., performed a study that evaluated YAP expression in colorectal cancer. This study revealed that YAP behaves as an oncogenic factor that promotes the generation of M2 TAMS through their polarization. In response to this process, the expression of tumorigenic phenotypes increases, from excess proliferation to the creation of CSC in the colon [110].

One of the reported critical issues in cancer is the increased anomalous expression of YAP/TAZ in the tumor microenvironment, leading to the recruitment of MDSCs to the tumor site. They form the premetastatic niche, disseminate the primary tumor, and inhibit the immune function, leading to tumor progression [111]. YAP plays a fundamental role in prostate cancer during the recruitment process because the YAP/TEAD complex induces the production of chemokine (C-X-C motif) ligand 5 and 6 (CXCL5, CXCL6), known as MDSCs regulators. The YAP/TAZ complex promotes cell migration and tumour cell binding to a suitable microenvironment. The complex is responsible for inducing immunosuppressive expression, which blocks immune cell responses [112]. Other mechanisms involve the secretion of IL-3, IL-4, IL-6, IL-17, IFN-γ, GATA3, Foxp3, and transforming growth beta factor 2 (TGFBR2). Those cytokines potentiate the immunosuppressed microenvironment ensuring the survival of the tumor [15,113,114,115].

### 5.5. Metabolic Reprogramming

Metabolic reprogramming is a tool used by tumor cells to satisfy their high nutrient demand by producing ATP and macromolecules for the biosynthesis of proteins and nucleotides. The goal is to promote tumor invasion, progression, and metastasis [116]. This process is influenced and regulated by YAP/TAZ since these work as nuclear and transcriptional mediators in different metabolic routes, promoting thesupress transcription of target genes that allow cells to adapt to the changing conditions of the tissue [117,118]. The role of these transcription cofactors in glycolysis, which is the main pathway used by tumor cells for sustained proliferation, stands out [119].

Enzo et al., reported that when YAP/TAZ activity is stimulated, the cell increases its glucose demand to promote glycolysis. At the same time, the inhibition of this process decreases the functions of the coactivators. Furthermore, it was observed that both phosphofructokinase 1 (PFK1) and 2 (PFK2) are instrumental for metabolic reprograming [120,121]. PFK1 binds the YAP/TAZ transcriptional cofactors to TEADs and this is the backbone of the regulation of the YAP/TAZ/TEAD complex’s stability. PFK1 is the crucial coordinator in this interaction because once its function is inhibited, the YAP/TAZ transcriptional module stops. This process favors transcription and specifically intensifies the malignant phenotype of tumor cells in breast cancer [120]. Multiple findings have corroborated that breast cancer tumors have increased expression of YAP/TAZ, which correlates to the argument that the majority of the genes involved are associated with glucose metabolism. This modification in cell metabolism results in a poor prognosis for patients with primary tumors [122,123].

Lipid metabolism can also modulate the transcriptional functions of YAP/TAZ [42]. Ye et al., observed that in non-alcoholic fatty liver disease associated with hepatocellular carcinoma, the release of free fatty acids leads to the expression of the junctional protein associated with coronary artery disease (JCAD). This protein couples with the LATS2 domain preventing the kinase from phosphorylating YAP, impeding its translocation into the nucleus, and activating the transcriptional module [124]. Furthermore, YAP potentiates its metastatic effects by changing from glycolysis to fatty acid oxidation during the metabolic reprogramming of tumor cells when dissemination to the lymph nodes occurs. Secondary tumors show an increased expression of genes involved in adipogenesis, fatty acid metabolism, oxidative phosphorylation, and cholesterol homeostasis [125]. The functions of YAP/TAZ in fat metabolism suggest that, during tumor progression, the regulation of the activity of both components is bidirectional. The ultimate goal is to meet the demand of cancer cells to favor their proliferation and later development [42].

**Figure 3:**
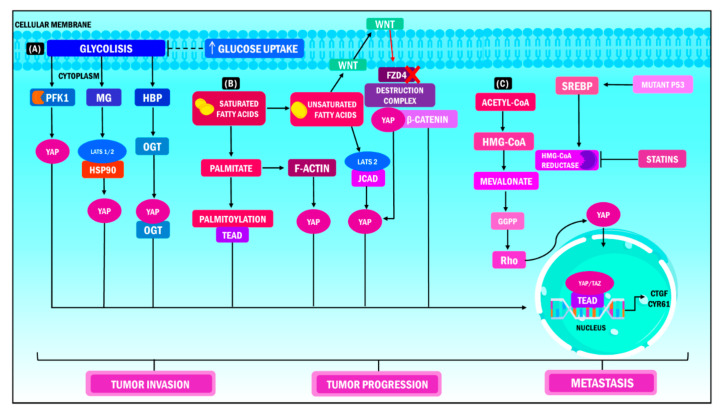
YAP/TAZ functions in metabolic heterogeneity. YAP/TAZ operate as nuclear and transcriptional mediators in different metabolic pathways during cancer, which results in increased tumor invasion, tumor progression and metastasis. (**A**) When YAP/TAZ activity is stimulated, the cell increases its glucose demand to promote glycolysis; the PFK1 enzyme is the coordinator of this interaction, considering that it regulates the stability of the union of the YAP/TAZ/TEAD complex in the cellular nucleus. (**B**) In terms of lipid metabolism, the release of free fatty acids induces the expression of the JCAD protein, while transcription of YAP occurs simultaneously through its dephosphorylation and union to the LATS2 domain. Consequently, translocation of YAP at the nuclear level, activation of the transcriptional module and transcription of target genes ensue. (**C**) The metabolism of mevalonate regulates the activity of YAP/TAZ through the production of geranyl pyrophosphate, which activates the Rho GTPase, which in turn dephosphorylates YAP/TAZ, promoting its accumulation in the cell nucleus and the transcription of its target genes. On the other hand, high levels of mevalonic acid boost the oncogenic activity of YAP /TAZ through the transcription of SREBP induced by the mutation of its cofactor p53. YAP: Yes-associated protein; TAZ: Transcriptional coactivator with PDZ-binding motif; PFK1: Phosphofructokinase 1; TEAD: Transcriptional enhanced associate domain; HBP: Hexosamine biosynthesis pathway; OGT: O-linked b-N-acetylglucosamine transferase; MG: Methylglyoxal; LATS: Large tumor suppressor kinase; HSP90: Heat shock protein 90; FZD4: Frizzled Class Receptor 4; JCAD: Junctional Cadherin 5 Associated; HMG-CoA: 3-hydroxy-3-methylglutaryl coenzyme A; GGPP: Geranylgeranyl pyrophosphate; SREBP: Sterol regulatory element binding protein; CTGF: Connective tissue growth factor; CYR61: Cysteine-rich angiogenic inducer 61.

There are additional metabolic pathways related to YAP/TAZ. Such is the case with the mevalonate pathway, which is known for being altered in multiple types of cancer. These include leukemia, lymphoma, multiple myeloma, breast cancer, liver cancer, pancreatic cancer, esophageal cancer, and prostate cancer. This pathway is related to synthesizing sterols and isoprenoids, critical molecules in tumor growth that are affected by statins [126,127]. Sorrentino et al., proposed that this pathway controls YAP/TAZ activity by producing geranyl pyrophosphate in breast cancer. This metabolite can activate Rho GTPases, and these enzymes dephosphorylate YAP/TAZ, promoting its accumulation in the cell nucleus and the transcription of its target genes. Likewise, increased levels of mevalonic acid in tumor cells promote YAP/TAZ oncogenic activity. This effect is caused by the enhanced transcription of sterol regulatory-element binding proteins (SREBP) induced by the mutation of its cofactor p53 (Figure 3) [128].

## 6. Resistance to Therapy: The Role of YAP and TAZ

**Figure 4:**
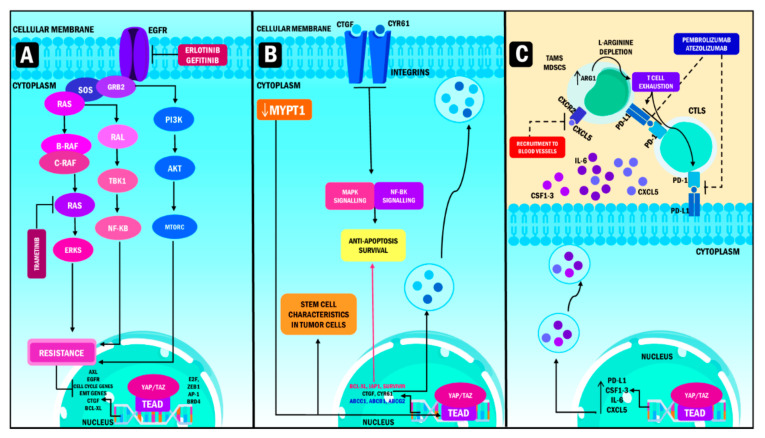
YAP/TAZ role in oncotherapy resistance. (**A**) Targeted therapy resistance. YAP/TAZ resistance to targeted therapy occurs mainly in EGFR and MAPK inhibitors, in non-small cell lung adenocarcinoma takes place thanks to the induction of high transcription levels of AXL. Meanwhile, YAP promotes resistance to MEK1/2 inhibition in neuroblastomas, with a hyperactive signaling of the *RAS* gene in cells treated with trametinib. Consequently, the overexpression of YAP produces transcription of genes that stimulate cell cycle entry, EMT, survival and cell death evasion, thus increasing resistance. (**B**) Chemotherapy resistance. The main mechanism used by YAP/TAZ to induce chemotherapy resistance is by binding to TEA domains, which produces the activation of the promoters of the *Cyr61/CTGF* oncogenes, causing resistance, evasion of apoptosis and hypoxia when overexpressed on the surface of tumor cells in breast cancer. In juxtaposition, the downregulation of MYPT1 produces the activation of the transcriptional module of the Hippo pathway, enhancing resistance to chemotherapy and the emergence of CSC phenotypes in ovarian cancer. (**C**) Immunotherapy resistance, PD-L1, CSF1-3, IL-6 and CXCL5 transcription create an enhanced immunosuppression activity by inhibiting monoclonal antibodies whose target is PD-L1. Subsequently, this phenomenon leads to the suppression of cytotoxic lymphocytes activity and a decrease in the production of cytokines resulting in T cell exhaustion. YAP: Yes-associated protein; TAZ: Transcriptional coactivator with PDZ-binding motif; TEAD: Transcriptional enhanced associate domain; MEK: Mitogen-activated protein kinase kinase; ZEB1: Zinc Finger E-Box Binding Homeobox 1; AP-1: activating protein-1; BRD4: Bromodomain Containing 4; CYR61: Cysteine-rich angiogenic inducer 61; MAPK: Mitogen-activated protein kinase; CXCR2: C-X-C Motif Chemokine Receptor 2; CXCL5: C-X-C Motif Chemokine Ligand 5; CSF1-3: Colony-stimulating factor 1–3; GRB2: Growth factor receptor-bound protein 2; TBK1: TANK Binding Kinase 1; NF-KB: nuclear factor kappa-light-chain-enhancer of activated B cells; PI3K: Phosphoinositide 3-kinases; AKT: Protein kinase B; MTORC: mammalian target of rapamycin complex; AXL: AXL Receptor Tyrosine Kinase; EGFR: Epidermal growth factor receptor; EMT: Epithelial to mesenchymal transition; CTGF: Connective tissue growth factor; BCL-XL: B-cell lymphoma-extra-large gene; MYPT1: Myosin Phosphatase Target Subunit 1; IAP1: Apoptosis inhibitor 1; ABCC1: ATP Binding Cassette Subfamily C Member 1; ABCB1: ATP binding cassette subfamily B member 1; ABCG2: TP Binding Cassette Subfamily G Member 2; PD-L1: Programmed death-ligand 1; CSF: colony stimulating factor; IL-6: Interleukin 6; PD-1: Programmed cell death protein 1; ARG1: Arginase 1; CTLS: Cytotoxic T lymphocytes; TAMS: Tumor-associated macrophages; MDSCS: Myeloid-derived suppressor cells.

An intrinsic mechanism of acquired resistance to different types of targeted therapy is the overexpression of YAP/TAZ [129]. The induction of resistance mediated by YAP/TAZ occurs by coupling these cofactors to TEAD and its potentiators, increasing the transcriptional activity of groups of genes that determine cell function and response. This event leads to a decrease in the effectiveness of anticancer therapies by reducing cancer cell sensitivity to these treatments. The goal is not achieved through the activation of growth signals, the promotion of progression in the cell cycle, apoptosis suppression, modulation of the response to DNA damage, initiation of EMT, and the induction of stem cell functions in cancer cells. The synergy of these processes causes the continued growth of the aggressive malignant phenotype of neoplastic cells (Figure 4) [129,130,131].

In this context, Nyungen et al., and Yi et al., describe three types of resistance according to the type of treatment. The first is represented by resistance to targeted therapy, which inhibits specific molecules and signaling pathways needed for the growth and survival of cancer cells. The researchers report that the overexpression of YAP/TAZ in cancer is an important type of resistance in this therapy, mainly in the context of the epidermal growth factor receptor (EGFR) and mitogen-activated protein kinase (MAPK) inhibitors [132,133]. Ghiso et al., performed immunohistochemical analysis of YAP/TAP in non-small lung adenocarcinoma cell lines resistant to EGFR tyrosine receptor kinase (TRK) inhibitors. They observed that all these cell lines had altered activation of YAP compared to those that did not exhibit resistance. At the same time, the authors showed that this phenomenon was caused by the induction of the receptor tyrosine kinase Axl, a known cause of resistance with anti-apoptotic effects when its expression is increased at the protein and RNA levels [134].

Coggins et al. performed RNA sequencing of cells treated with trametinib, an inhibitor of Mitogen-activated protein kinase kinase 1/2 (MEK1/2) which delays cell growth. The authors showed that YAP promotes the resistance of neuroblastoma cells to MEK1/2 inhibitors by promoting RAS signaling [135]. It was reported that the drug caused the nuclear translocation of YAP due to the decrease in its phosphorylation, showing that cell resistance to trametinib is directly proportional to the levels of YAP in them. Therefore, its overexpression causes resistance, while depletion causes the arrestment of the cell cycle G1/S mediated by the lack of MYC/MYCN and E2F.

The second type of resistance corresponds to chemotherapy resistance, a treatment that interferes with cell division and survival. Recent analyses have reported a relationship between YAP/TAZ and therapeutic resistance [132]. Lai et al., described that, in its oncogene function, increased levels of TAZ are responsible for Taxol (paclitaxel) resistance in breast cancer. Likewise, it was identified that resistance occurs through the union of TAZ to the transcription factors of the TEAD family, which activate the promoters of oncogenes cysteine-rich angiogenic inducer 61 / connective tissue growth factor (*CYR61*/*CTGF)*. When these oncogenes are overexpressed in breast epithelial cells they cause resistance to the antimitotic effect through apoptosis and hypoxia evasion. These findings show that TAZ signaling is an essential modifier of the response to chemotherapy in breast cancer cells [136].

In addition, Muñoz et al., analyzed myosin phosphatase target subunit 1 (MYPT1) expression levels in samples of ovarian tumor tissue. The results showed that MYPT1 is downregulated in tumors, which causes the activation of the transcriptional module of the Hippo pathway, specifically YAP. This effect potentiates the stem cell characteristics in ovarian cancer cells, leading to resistance to cisplatin chemotherapy. Furthermore, the same study showed that the cells responded to the treatment [137]. It is essential to consider pharmacological therapies that can block YAP/TAZ expression to obtain the expected response in certain types of chemotherapy according to the type of cancer.

Finally, the third type of resistance corresponds to resistance to immunotherapies. The transcription and expression of PD-L1 and the secretion of colony-stimulating factor 1-3 (CSF1-3), IL-6 and CDCL5 create an immunosuppressive network and the inhibition of PD-L1 signaling pathway after treatment with monoclonal antibodies (atezolizumab, durvalumab) or inhibition of the PD-1 receptor (pembrolizumab, nivolumab) [132]. Kim et al. performed a study in which the researchers observed that the expression of PD-L1 induced by YAP promotes the evasion of the immune response in melanoma. Likewise, it was reported that YAP expression is directly linked to PD-L1 expression, increasing the suppression of the activity of the cytotoxic lymphocytes or TCD8, as well as a decrease in cytokine production [138]. It is suggested that when YAP/TAZ expression is elevated, immunosuppression is observed, which is characteristic of malignant neoplasias. Therefore, cancer cells can show decreased response to immunotherapy with later resistance to treatment.

## 7. YAP and TAZ as Therapeutic Targets

The biology and regulation of YAP/TAZ have gained popularity for their crucial role in cancer development and progression. Consequently, they have become targets for developing potential therapies that inhibit their effects [139,140] (Table 1). Different drugs can directly or indirectly hinder the coactivators’ oncogenic activities. These drugs significantly contribute to the administration of more effective anticancer treatments [141].

The US Food and Drug Administration (FDA) has approved verteporfin (VP), a small molecule derived from benzoporfirin that inhibits YAP/TAZ [147], for the treatment of age-related macular degeneration [148]. VP is a light-sensitive molecule initially developed to inhibit tumor growth and development [149]. Recent in vitro studies show that this drug has biological functions independent of its activation through light exposure. One of these is the direct inhibition of YAP expression and its interaction with the TEAD to avoid the transcription of its target genes [142].

Gibaut et al. [150] showed that VP significantly inhibits the luciferase activity of TEAD, causing a decrease of YAP/TAZ expression across MDA-MB-231 cell lines in breast cancer. This phenomenon is related to reducing the cotranscriptional activity of the YAP/TAZ/TEAD complex. VP could induce oligomers of great molecular weight through the cross-link of proteins in the presence of oxidative stress. It has been suggested that this process would be directly related to inhibiting YAP/TAZ activity. However, its multimeric form was not detected, concluding that VP inhibits its degradation and/or modification [150]. Nonetheless, the exact mechanism and molecules involved in this process continue to be unknown.

Data collected from endometrial cancer tissues revealed that VP induces the cytoplasmic retention of YAP and, indirectly, TAZ through the increase in the levels of 14-3-3σ protein. Likewise, VP increases p53 levels, an instrumental process for the potentiation of its effect. The rise in this tumor suppressor protein is directly proportional to the levels of 14-3-3σ [151].

It has also been observed that VP can increase sensitivity and combat resistance to anticancer treatments by decreasing YAP expression. Shi et al., showed that VP treatment could reverse taxol resistance caused by YAP overexpression in the LOVO/TAC cell lineage. Likewise, combined therapy with these two drugs showed a more significant inhibitory effect in the expression of this oncoprotein. These results show the potential of VP as an effective YAP/TAZ inhibitor [152], and it was established that it uses multiple pathways to stop YAP/TAZ expression. These include the blockage of the YAP/TAZ/TEAD correlation, YAP/TAZ cytoplasmic arrest, and/or the potentiation of the anticancer effects of other treatments.

Other molecules have been identified as important YAP/TAZ inhibitors. Dasatinib, a second-generation tyrosine kinase inhibitor, is commonly used to treat chronic myeloid leukemia [153]. Sun et al., determined that this drug acts on the Src kinase, causing the activation of the JNK-L1MD1-LATS cascade. This cascade leads to the inhibition of the transcriptional program mediated by YAP, related to its oncogenic function. This study proposes a novel therapeutic alternative to target YAP expression through its interaction with Scr, which acts as an upregulator of the YAP pathway [143].

Statins, used as standard treatment against hypercholesterolemia, have been known to inhibit the YAP/TAZ pathway. The inhibition of the hydroxymethylglutaryl-coenzyme A (HMG-CoA) reductase and the mevalonate pathway have been demonstrated to regulate YAP activation [154]. Statins may affect YAP nuclear localization by inducing phosphorylation, cytoplasmic retention, degradation, and the target genes’ decreased transcription by TEA domains [155]. A study carried out by Fang et al. in pancreatic cancer cells demonstrated the mechanism of simvastatin and cerivastatin inhibition on these oncoproteins. In particular, the therapy induces YAP relocalization from the nucleus to the cytoplasm. It inhibits the expression of the CTGF, CYR61, and Baculoviral IAP Repeat Containing 5 (Birc5), as well as the blockade of colony formation potential on these cells [144]. Therefore, they have a critical role in preventing tumor invasion and metastasis [156].

An in vitro study performed by Ha et al., showed the impact of different statins in YAP location and their activity in ductal pancreatic adenocarcinoma cells. Lipophilic statins, cerivastatin, and simvastatin inhibit YAP nuclear translocation, transcriptional activity, and phosphorylation. These findings show that the use of statins in oncology should be scrutinized [144]. Likewise, the simultaneous administration of multiple inhibitors has proved to be effective for targeting the effects of YAP/TAZ. Oku et al. [141] reported that the combination of dasatinib, statins, and pazopanib inhibited the nuclear translocation of YAP/TAZ in breast cancer cells. The inhibition was due to the phosphorylation of the complex and proteasomal degradation by pazopanib. Furthermore, it was established that combining these drugs with other anticancer therapies can increase response to treatment in neoplastic cells and arrest YAP/TAZ-dependent breast cancer development [141].

New studies have highlighted the role of the topoisomerase inhibitor A35, synthetic berberine, on tumor proliferation. The compound decreased the expression of YAP/TAZ and its target genes. It phosphorylates the Ser127 residue of YAP, then it binds to TAZ and retains both in the cytoplasmic form, disrupting their presence in the nucleus. A35 can induce cell cycle arrest in the G2/M phase. This effect is susceptible to YAP levels since the increased expression of this protein is sufficient to decrease the transcription of genes and pathways associated with the G2/M phase arrest [145,157].

A new method to inhibit YAP/TEAD interaction and the transcription of its target genes was described by Jiao et al., A peptide known as “Super-TDU”, which simulates the functions of VGLL4, a transcriptional cofactor described as a tumor suppressor, is used [158]. The peptide competes with YAP in the binding to TEA domains, inhibiting the oncogenic activity of YAP. When this peptide was administered to mice with stomach cancer, a YAP/TEAD complex was observed along with a decreased expression of the genes *CTGF*, *CYR61*, and Caudal Type Homeobox 2 (*CDX2*). This inhibition stopped the formation and growth of malignant cells [146].

Additional molecules have been reported as potential therapeutic options [10]. They are an extensive research subject in cancer therapy because of the vast possibilities of inhibiting YAP/TAZ CSC. However, the extent of the therapeutic effects is still unknown [27,159]. Clinical assays are required to evaluate the validity and usefulness of YAP/TAZ inhibitors and the degree of toxicity they could have.

Although YAP/TAZ function is often expendable in adult homeostasis, both cofactors are dynamic and are involved in several cellular processes. Different experimental studies have shown that the inhibition of these coactivators causes adverse effects in adult mice. Among these adverse effects are (1) a decrease in smooth muscle cells growth and proliferation at the cardiovascular level [160], (2) colonic pseudo-obstruction [161], (3) decrease of kidney function with the development of proteinuria and progressive kidney failure [162], (4) progressive decrease in the levels of steroidogenic genes of the suprarenal cortex [163], (5) lung hypoplasia, (6) hepatomegaly, and (7) other findings [164,165]. Therefore, it is necessary to perform continued research into the underlying mechanisms that give these coactivators their functions as oncogenes. Similarly, it is essential to determine each drug’s specific mechanism of action as inhibitors of YAP/TAZ functions to identify their true potential and success. This way, we will be one step closer to clearing the inherent doubts associated with YAP/TAZ treatment in the fight against cancer.

## 8. Conclusions

YAP/TAZ molecules are known transcriptional coactivators that promote the transcription of target genes in different signaling pathways that regulate cell proliferation and apoptosis under physiologic circumstances. The influence of these molecules in numerous systemic processes is evident, as is the relationship between the anomalous expression of YAP/TAZ in pathological phenomena such as cancer.

The primary role of both coactivators in different molecular pathways allows them to regulate different essential aspects for neoplastic development, such as cell proliferation, plasticity, and cancer cell growth and survival. This takes place through different events, including angiogenesis, immune evasion, cell metabolism, EMT, mechanotransduction, cell cycle control, mitosis, and DNA repair. Therefore, these molecules have an essential role in cancer progression.

Consequently, the therapeutic potential of YAP/TAZ inhibition has been proposed as a novel alternative in a wide range of anticancer treatments, with different clinical trials currently being performed. Other YAP/TAZ inhibitor drugs have proven to have antitumor effects individually or as potentiators of combined therapies. However, to determine the feasibility of these emergent therapeutics, it is necessary to fully identify the molecular mechanisms, the functions of YAP/TAZ during carcinogenesis, and the toxicity levels that could be generated by inhibiting the complex.

## Figures and Tables

**Table 1:** Molecules targeting YAP/TAZ in cancer therapy-preclinical evidence.

Molecules	Exp. Model	Duration	Relevant Results	References
Verteporfin	OVCAR8 xenograft mice.	3 weeks	In vivo, VP significantly affected tumor growth in OVCAR8 xenograft mice, resulting in tumor nodules with lower average weight and reduced volume of gross ascites.	[142]
Dasatinib	Four-to six-week-old nu/nu athymic BALB/c female mice.	3 weeks	Dasatinib can impair renal carcinoma cell viability in vitro and decrease tumor growth in vivo.	[143]
Lipophilic statins	PANC-1 and MiaPaCa-2LSL-human pancreatic cancer cell lines, and KrasG12D/+ and p48-Cre+/− mice.	2 weeks	Lipophilic statins limit YAP activity and proliferation in pancreatic cancer cell models in vitro and they attenuate early lesions that lead to pancreatic ductal adenocarcinoma in vivo.	[144]
Dasatinib, statins and pazopanib	MDA-MB-231, MDA-MB-453, HBC-4, HBC-5, MCF-7, BSY-1, ZR-75-1, and SKBR-3 breast cancer cell lines.	2 weeks	All drugs induced phosphorylation of YAP and TAZ, and pazopanib induced proteasomal degradation of YAP/TAZ.	[141]
A35	Human K562, HepG2, Raji, HCT116 and HCT116-KO cancer cells.	1 week	A35 decreased YAP1 nuclear localization by activating YAP phosphorylation (Ser127), which subsequently regulated the transcription of YAP target genes associated with growth and cycle regulation to induce G2/M arrest and growth inhibition.	[145]
Super-TDU	Human gastric tumor clinical specimens.	-	Super-TDU inhibited cell viability and colony formation of GC cell lines MGC-803, BGC-823, and HGC27. This peptide downregulated the expression of YAP-TEADs target genes *CTGF*, *CYR61*, and *CDX2*.	[146]

Abbreviations: YAP: Yes-associated protein; TAZ: Transcriptional coactivator with PDZ-binding motif; TEAD: Transcriptional enhanced associate domain; VP: verteporfin, GC: gastric carcinoma; CTGF: Connective tissue growth factor; CYR61: Cysteine-rich angiogenic inducer 61; CDX2: Caudal Type Homeobox 2.

## Data Availability

Not applicable.
